# ZnO@TiO_2_ Core Shell Nanorod Arrays with Tailored Structural, Electrical, and Optical Properties for Photovoltaic Application

**DOI:** 10.3390/molecules24213965

**Published:** 2019-11-01

**Authors:** Ivana Panžić, Krunoslav Juraić, Nikša Krstulović, Ana Šantić, Domagoj Belić, Damjan Blažeka, Milivoj Plodinec, Vilko Mandić, Jelena Macan, Adnan Hammud, Danail Ivanov, Jasper Plaisier, Marc Gregor Willinger, Davor Gracin, Andreja Gajović

**Affiliations:** 1 Ruđer Bošković Institute, Bijenička cesta 54, 10000 Zagreb, Croatia; ipanzic@irb.hr (I.P.); kjuraic@irb.hr (K.J.); asantic@irb.hr (A.Š.); dbelic@irb.hr (D.B.); mplodinec@fhi-berlin.mpg.de (M.P.); gracin@irb.hr (D.G.); 2 Institute of Physics, Bijenička cesta 46, 10000 Zagreb, Croatia; niksak@ifs.hr (N.K.); dblazeka@ifs.hr (D.B.); 3 Fritz-Haber-Institut der Max-Planck-Gesellschaft, Faradayweg 4-6, 14195 Berlin, Germany; hammud@fhi-berlin.mpg.de (A.H.); ivanov@fhi-berlin.mpg.de (D.I.); marc.willinger@scopem.ethz.ch (M.G.W.); 4 Faculty of Chemical Engineering and Technology, University of Zagreb, Marulićev trg 19, 10000 Zagreb, Croatia; vmandic@fkit.hr (V.M.); jelena.macan@fkit.hr (J.M.); 5 Sincrotrone Trieste, Strada Statale 14, km 163.5, 34012 Basovizza (TS), Italy; jasper.plaisier@elettra.eu; 6 ETH Zürich, Auguste-Piccard-Hof 1, 8093 Zürich, Switzerland

**Keywords:** core–shell, ZnO nanorods, TiO_2_ thin film, pulsed laser deposition, DC reactive magnetron sputtering, chemical deposition, electrical properties, optical properties

## Abstract

ZnO has prominent electron transport and optical properties, beneficial for photovoltaic application, but its surface is prone to the formation of defects. To overcome this problem, we deposited nanostructured TiO_2_ thin film on ZnO nanorods to form a stable shell. ZnO nanorods synthesized by wet-chemistry are single crystals. Three different procedures for deposition of TiO_2_ were applied. The influence of preparation methods and parameters on the structure, morphology, electrical and optical properties were studied. Nanostructured TiO_2_ shells show different morphologies dependent on deposition methods: (1) separated nanoparticles (by pulsed laser deposition (PLD) in Ar), (2) a layer with nonhomogeneous thickness (by PLD in vacuum or DC reactive magnetron sputtering), and (3) a homogenous thin layer along the nanorods (by chemical deposition). Based on the structural study, we chose the preparation parameters to obtain an anatase structure of the TiO_2_ shell. Impedance spectroscopy shows pure electron conductivity that was considerably better in all the ZnO@TiO_2_ than in bare ZnO nanorods or TiO_2_ layers. The best conductivity among the studied samples and the lowest activation energy was observed for the sample with a chemically deposited TiO_2_ shell. Higher transparency in the visible part of spectrum was achieved for the sample with a homogenous TiO_2_ layer along the nanorods, then in the samples with a layer of varying thickness.

## 1. Introduction

Zinc oxide has been applied in many fields; including gas sensors, photodiodes, lithium-ion batteries and particularly solar cells. ZnO can be easily fabricated into various morphologies (e.g., nanowires, nanorods, nanotubes, nanoflowers, nanosheets) on different substrates. Therefore, ZnO in different shapes and in composites is studied for various applications. Recently, some authors combined ZnO in heterostructure with CdS [1,2] and studied its photocatalytic properties. ZnO in the form of thin film with CdS nanoparticles on the surface has been shown to be a high-performance material for photocatalytic degradation of methylene blue [1], while ZnO/CdS hierarchical composites have shown great photocatalytic H_2_-production performance [2]. Wu et al. [3] prepared ZnO hollow spheres and the hedgehog-like ZnO hollow spheres and studied their use as anodes for lithium-ion batteries, having good rate capacity, cycling performance, and high initial specific capacity. ZnO nanowires have prominent electron transport properties [4], which are beneficial for solar cell application. However, ZnO is not chemically stable and is prone to dissolution. One way to avoid this fact is to coat a chemically stable shell on ZnO nanowires [5].

On the other hand, nanostructured titanium dioxide is known as a material with notable physical and chemical properties, such as high photo-activity and environmental stability, as well as low cost synthesis. TiO_2_ exists in nature in three different crystalline phases: anatase, rutile, and brookite, but it was found that anatase (band gap ~3.2 eV) is more photo-active than other modifications. It is widely used for photovoltaic applications [6], primarily as an electron transport layer (ETL) in perovskite and dye sensitized solar cells, due to a suitable band gap for the acceptance of electrons from the active layer of solar cells. The studies aimed to improve the optical and transport properties of TiO_2_-based electron conduction layers, which are of considerable importance for the enhancement of photovoltaic performance. By a combination of the high reactivity of TiO_2_ and the large binding energy of ZnO, the process of electron transfer between the corresponding conduction and valence bands can be facilitated in the composite system [7].

In comparison to mesoporous thin films on transparent conductive oxide (TCO) operated as ETL in solar cells, 1-dimensional (1D) nanostructures on TCO substrates can provide a direct pathway for electron transport and offer open channels for filling with light absorbers and HTMs for solar cells. Moreover, oriented nanorod-like materials on TCO are known for their efficient charge separation and transport properties and are thus favorable for achieving good device performance [8,9,10]. Therefore, growth of oriented nanorods (NRs) on TCO substrates has drawn extensive research interest in solar energy utilization in the past years.

Moreover, the utilization of the single-crystalline 1D nanostructures with preferred orientation for ETL decreases the number of grain boundaries that decrease conductivity, as was observed in studies on ZnO nanostructure [11,12,13]. Thus, Wu et al. [11] investigated rapidly synthesized ZnO nanowires for photodetector application and produced nanowires which consist of nanoparticles. They concluded that these nanowires have adsorption sites on the inter-granular surface, which can lead to the increased reactivity of these surfaces and, therefore, a low conductivity. Their finding is in accordance with old paper of Oktik [12] where he stated that a large density of extrinsic electron traps, present at the grain boundaries due to oxygen chemisorption, results in a barrier to charge-carrier conduction at grain boundaries. Annealing in vacuum or in reducing atmosphere desorbs the oxygen and therefore decreases the grain boundary potential barrier and increases the effective charge-carrier concentration (and therefore the conductivity). Natsume and Sakata [13] prepared ZnO films by sol–gel spin-coating and confirmed the grain boundary scattering effect due to thermionic emission.

Several groups prepared ZnO nanowires combined with other materials (polycrystalline MgO, P3HT polymer) in core–shell hetero-structures and studied their application in polymer and dye sensitized solar cells (DSSCs) [14]. These hetero-structures combine stable characteristics from the shell and fast electron transport features of the mono-crystalline nanowire core, thus increasing the efficiency of solar cells. Samad et al. [15] found that an enhancement of photocurrent density and photo conversion efficiency occurred due to the sufficient Ti content within TiO_2_–ZnO nanorod films. This film traps the photo-induced electrons and minimizes the recombination of charge carriers, while the charge-separation effect at the type-II band alignment of Zn and Ti further enhances the charge carrier transport during illumination. Boroa et al. [16] reviewed different TiO_2_/ZnO nanocomposites for use as a photo-anode in DSSCs. They compared typical solar cells parameters (open circuit voltage, short circuit current, fill factor and efficiency) for nanocomposites such as TiO_2_ doped or decorated with different sized ZnO nanostructures and vice versa, coaxial nanotube arrays, nanodonuts, nanoflowers and 3D hierarchical hetero-structures. It is concluded that the best performance among studied nanostructures was shown in DSSCs with one-dimensional composite structures such as nanotubes, nanorods, or nanowires. Recently, Zhong et al. [17] synthesized ZnO nanorod arrays by polymer template and sol–gel methods for TiO_2_ modification, starting from butyl titanate and isopropanol. They studied the use of such core–shell structures as ETL in perovskite solar cells (PSCs) and showed improved power conversion efficiency compared to pure ZnO nanorod arrays used as ETL. Enhanced stability in air was also observed. The same authors [18] also studied the influence of the ZnO–TiO_2_ nanorod length, prepared by the same procedure on PSCs efficiency and stability. All the cited results for photovoltaics with ZnO–TiO_2_ composites as ETL indicated that the study of the influence of the TiO_2_ deposition parameters on structure, morphology, and, consequently, on optical and electrical properties are of the main importance for further development of different photovoltaic devices. Therefore, detailed study of different preparation methods and the influence of the preparation parameters of the TiO_2_ shell in ZnO@TiO_2_ core–shell nanostructures would improve photovoltaic devices based on ZnO@TiO_2_ core shell nanorod arrays as ETL.

ZnO with TiO_2_ at the surface of the nanostructures are also used for other applications. Hernandez et al. [19] have demonstrated that ZnO–TiO_2_ materials have a superior photo-electrochemical activity, due to improved electron-hole separation and lower charge carrier recombination rates. They demonstrated a sol–gel synthesis for the deposition of a protective shell of anatase TiO_2_ nanoparticles onto a vertically aligned ZnO nanowire (NW) array. It is reported that the presence of the TiO_2_ shell in the core–shell nanostructure improves the charge separation efficiency, thanks to a potential barrier at the semiconductor/electrolyte interface, which limits the charge recombination. Therefore, in ZnO@TiO_2_ core–shell nanostructures, the high electron mobility is coupled with the efficient separation of charge carriers between the TiO_2_ shell and the ZnO core. Further, X. Yan et al. [20] applied the heterogeneous nanostructure as an efficient way to improve light absorption and extend the carrier life time characteristics of the photocatalysts to extend the catalyst’s optical absorption range in a Zn/TiO_2_ core-brush structure.

In this work, we focused on influence of the type of preparation technique and deposition parameters on the structure and morphology of ZnO@TiO_2_ core–shell hetero-structures and their correlation with optical and electrical properties. The aim was to achieve a simple and low-cost preparation process of ZnO@TiO_2_ core–shell nanostructures and to understand their electrical and optical properties intended for photovoltaic application. However, the essential goal was to preserve transparency and to obtain superior electron transport properties of the formed ZnO@TiO_2_ core–shell nanorod array layers, so different deposition techniques for preparation of theTiO_2_ layers were studied. The ZnO nanorod arrays were prepared by a simple chemical method, while for TiO_2_ deposition on the ZnO nanorods, three different methods were studied—pulsed laser deposition (PLD), magnetron sputtering (MS), and spin coating of a chemically prepared precursor for the TiO_2_ layer, followed by annealing. The morphology and crystal structure of the deposited TiO_2_ is considerably dependent on the deposition method. The preparation parameters were chosen to obtain an anatase structure of the TiO_2_ shell. A significant absorption in the UV part of the spectrum was observed for all the prepared morphologies, while the absorption of visible light depends on the morphology of the TiO_2_ layer. The superior electrical properties in comparison to uncovered ZnO nanorods and bare TiO_2_ layers were observed in the thin layer of ZnO@TiO_2_ core–shell nanostructures. Thus, high surface conductivity for electrons as charge carriers as well as low activation energy was observed for all the core–shell nanostructures. The best electrical and optical properties among the studied samples were obtained for core–shell nanostructures where the TiO_2_ layer was prepared by chemical deposition, which was explained by homogenous coverage of the ZnO nanorods as well as by the smoothness of the TiO_2_ surface.

## 2. Results

### 2.1. Structural Results

#### 2.1.1. ZnO Nanorods

ZnO nanorods (ZNRs) were prepared on an indium tin oxide (ITO) coated glass substrate to study the growth of these layers and nanorods on ITO. For the electrical measurements, the nanorods were also grown on a glass (quartz) substrate to avoid the possible influence of ITO on the ZNR electrical response.

**Figure 1:**
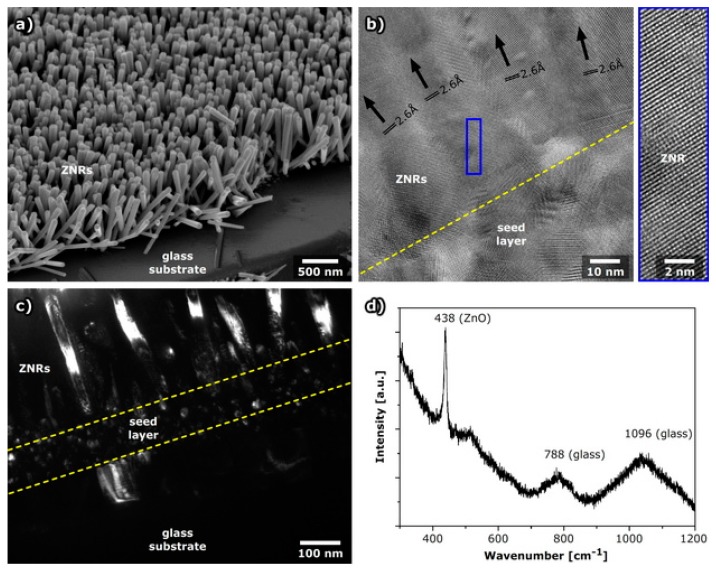
ZnO nanorods prepared on glass substrate: (**a**) SEM image, (**b**) HRTEM image. The d-spacings of the (002) plane are indicated for every nanorod, enlarged image of the crystal planes are shown separately, (**c**) dark field TEM image using (002) orientation, (**d**) Raman spectrum of ZNR on glass substrate. The position of the Raman active ZnO band as well as broad bands of glass substrate are indicated above the spectrum.

It was observed by scanning electron microscopy (SEM) that nanorods grow homogenously and their length varies 10% to 20% (estimated from Figure 1), as recorded at the edge of the tilted sample. A cross section of the nanorods observed by high-resolution transmission electron microscopy (HRTEM) and dark field TEM shows the preferential growth of the nanorods that start the growth from nanoparticles in the seed layer (Figure 1b,c). The d-spacing observed in nanorods by HRTEM was around 0.260 nm which corresponds to the (002) plane of zincite, hexagonal ZnO (JCPDS PDF#36-1451). Raman spectroscopy also shows a band characteristic for hexagonal ZnO at 438 cm^−1^ [21,22], as well as broad bands of glass substrate.

#### 2.1.2. TiO_2_ Shell Layer Prepared by Pulsed Laser Deposition (PLD)

The influence of the PLD parameters on the morphology and structure of formed TiO_2_ layers was studied with the aim to achieve ZnO@TiO_2_ core–shell nanostructures transparent to visible light. The number of laser pulses as well as atmosphere (argon or vacuum) used for PLD deposition was optimized based on comprehensive study of the TiO_2_ layer obtained at the surface of the ZNR using different characterization techniques. Here we show the main results for the prepared samples further studied by the optical and electrical measurements. The rest of the results obtained during parameter optimization process are shown as supplemental materials.

**Figure 2:**
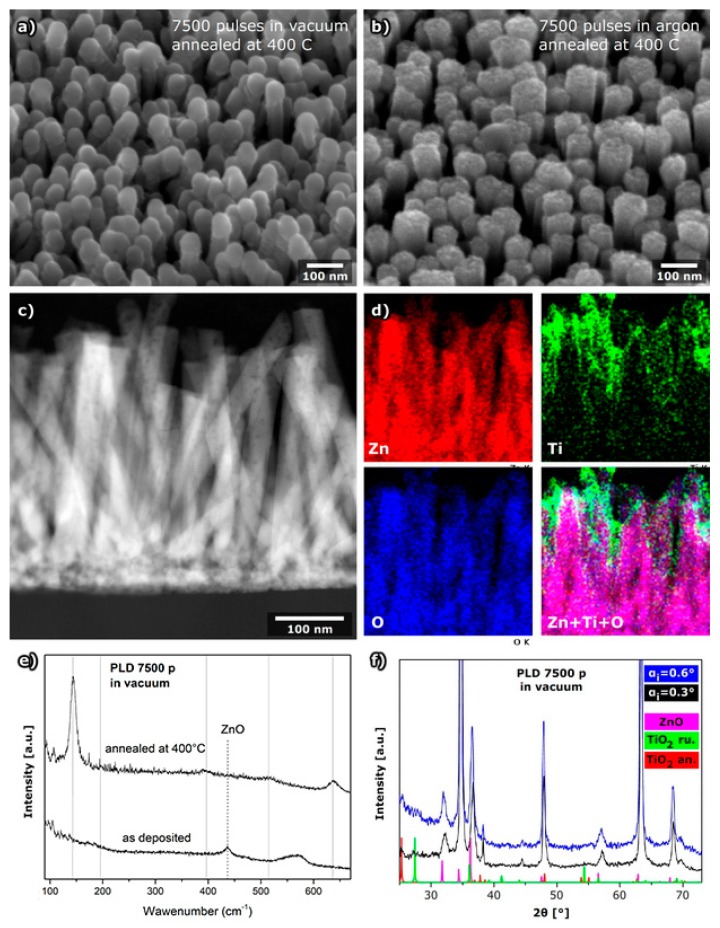
ZNRs with a layer of TiO_2_ deposited by PLD. (**a**) SEM image of sample deposited in vacuum by 7500 pulses of laser (5 Hz) after heating in air at 400 °C (side view), (**b**) SEM image of sample deposited in argon with 7500 pulses of laser (5 Hz) after heating in air at 400 °C (side view), (**c**) scanning transmission electron microscopy (STEM) image of cross section of sample deposited in a vacuum, (**d**) energy dispersive X-ray spectroscopy maps of zinc, titanium, oxygen, and the distribution of all the elements in nanorods, (**e**) Raman spectra before and after annealing (vertical lines denote position of anatase bands) and (**f**) grazing incidence X-ray diffraction of sample prepared by PLD applying 7500 pulses in a vacuum.

For the ZNR sample with a deposited TiO_2_ layer using 7500 laser pulses (5 Hz repetition rate) in a vacuum, a smooth surface of TiO_2_ at the ZNR was formed in a hemisphere-like shape on the top of the nanorods, as was presented in Figure 2a. On the other hand, TiO_2_ deposited in Ar atmosphere (30 Pa) formed nanoparticles and clusters on the surface of ZNRs (Figure 2b).

It was found before for other materials that films deposited in a vacuum or at low working gas pressure are compact with a wide variation in the size of the columnar structures in the film, while those deposited at higher pressures of Ar are rather rough with more spherical particles [23]. When laser ablation is performed in a vacuum, ablated particles (plasma plume) follow free expansion. When laser ablation is performed in the background gas, plasma plume is confined (collision dominated regime) and thus decelerated [24]. In that case, velocity of ablated particles decreases with pressure [25] while particle expansion can be described either with drag-force or a shockwave model [26]. Ablated particles have also a time-of-flight character of expansion as lighter and ionized particles are faster than heavier or neutral ones [27]. However, the kinetics of ablated particles highly influence the formation processes of thin films fabricated by the PLD technique. Higher energies of ablated particles lead to longer surface diffusion lengths when they reach the substrate. It is well known that longer surface diffusion length of atoms results in smoother, more compact, and denser layers as the higher mobility of deposited atoms increases the probability of smoothing the ridges on the growing surface front [28,29]. Atoms with low surface mobility (ablated particles with low velocities) are frozen at place where they impinge the substrate and they lead to a formation of a rough layer. That is why the layer of TiO_2_ deposited by PLD in Ar is rough while that formed in a vacuum is smooth.

To determine the amount of deposited TiO_2_ by different deposition routes, energy dispersive X-ray spectroscopy (EDS) using SEM was performed at a larger area of the samples. It was observed that in samples prepared by PLD in a vacuum, a much higher amount of TiO_2_ was deposited (Table 1) then in the case when deposition was done in an Ar atmosphere.

To verify the distribution of TiO_2_ on the surface of the nanorods, dark field STEM and EDS mapping was done using an atomic resolution transmission electron microscope (Figure 2c,d). It was observed that even if the TiO_2_ layer is applied in a vacuum to form a smooth surface of TiO_2_, the ZNR are not homogeneously covered by TiO_2_, but the layer of the TiO_2_ is thicker at the upper part of the nanorods.

With the aim to determine the crystal structure of the formed TiO_2_ layer confocal micro-Raman measurements were done. It was shown that the deposited TiO_2_ was amorphous regardless of deposition parameters (Figure 2e and Figure S2) since in as-deposited samples only the band characteristic of zincite was observed. Therefore, all the samples were annealed after deposition to obtain crystalline TiO_2_, but the SEM images of the as-deposited samples and the annealed samples indicated that post-deposition sample heating did not influence the morphology of TiO_2_ (Figure S1). Since it is known that the TiO_2_ anatase phase has better optical properties than the rutile phase, the parameters of deposition and heating temperature were optimized to obtain a pure anatase nano-layer. The layers deposited in a vacuum by 7500 pulses and annealed at 400 °C clearly showed only anatase structure of TiO_2_ by Raman spectroscopy and a band of ZnO of very low intensity (Figure 2e). In the sample deposited in a vacuum, even after annealing at 450 °C, the anatase structure dominated, with just one very low intensity band characteristic for the rutile phase (Figure S2a). However, the phase transition to rutile was already observed after heating at 400 °C in samples prepared by PLD in Ar (Figure S2b), although it is known that the phase transition from anatase to rutile is very slow for temperatures under 900 °C. This behavior can be explained by the formation of small nanoparticles at the sample surface (Figure S1) in the case of PLD performed in Ar. Since a smaller amount of TiO_2_ was deposited in Ar (Table 1) by 5000 pulses, in this case TiO_2_ was not detected by Raman spectroscopy (Figure S2b). The absence of a rutile phase in samples prepared in a vacuum can be explained by the formation of a continuous TiO_2_ thin layer (Figure 2 and Figure S1) which prevents the formation of rutile.

The samples obtained by PLD using 7500 pulses in a vacuum were additionally studied by grazing incidence X-ray diffraction (GI-XRD; Figure 2f) to confirm the Raman spectroscopy results on the structural phases of TiO_2_ as well as their distribution at the ZNR. Two grazing incidence angles were used. The first one, close to critical angle (α_I_ = 0.3), provides information about the surface while other one (α_i_= 0.6), where probe beam penetrates deeper in the sample, enables analysis of the “bulk” of the sample. The peaks characteristic for hexagonal ZnO (JCPDS PDF#36-145) are dominant in diffraction patterns recorded at both angles, while the diffraction lines of the TiO_2_ anatase phase were detected only at an angle of 0.3°. This result implies that the majority of TiO_2_ in the ZnO@TiO_2_ core–shell nanorod arrays was deposited on the upper part of the nanorods. That result agrees with EDS mapping observations (Figure 2d).

#### 2.1.3. TiO_2_ Shell Layer Deposited by Magnetron Sputtering

**Figure 3:**
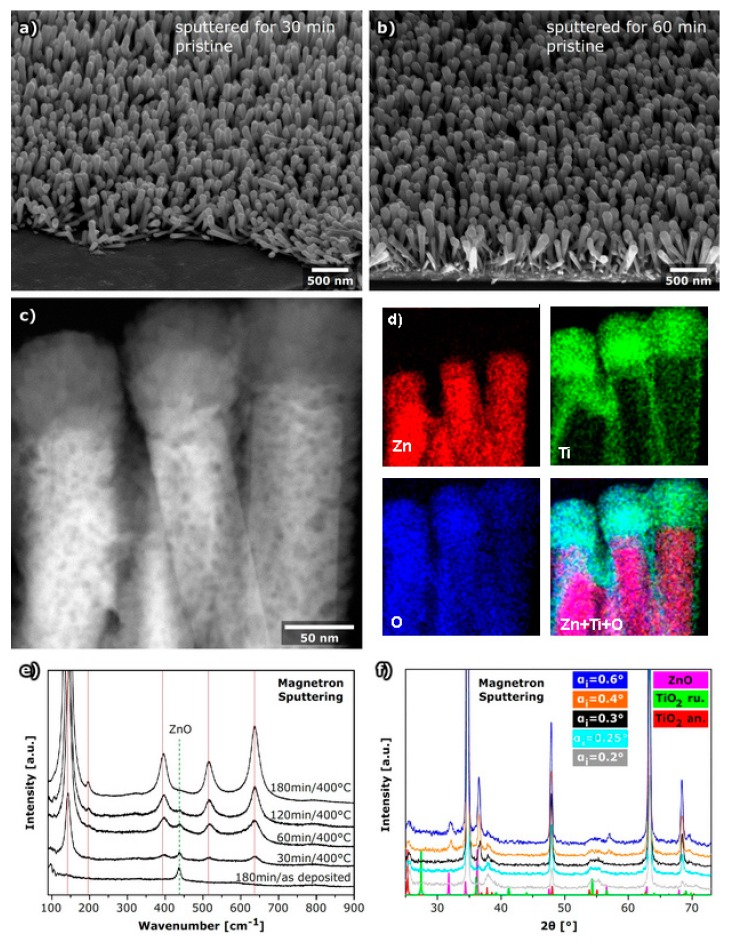
ZNRs with a layer of TiO_2_ deposited by DC reactive MS. (**a**) SEM image after 30 min of deposition, (**b**) SEM image after 60 min of deposition, (**c**) STEM dark field image of a cross section of a sample deposited after 60 min, (**d**) EDS maps of zinc, titanium, oxygen, and the distribution of all the elements at nanorods, (**e**) Raman spectra before and after annealing for samples deposited after different deposition time denoted above spectra (vertical lines denote position of anatase bands) and (**f**) GI-XRD of sample prepared in 60 min of deposition.

In DC reactive magnetron sputtering experiments, the deposition time was varied with the aim to find appropriate parameters to obtain a transparent ZnO@TiO_2_ core-shell nanorod array that will have good electrical properties. The SEM study indicated that after 30 min of deposition, a very thin layer of TiO_2_ was deposited on the nanorods (Figure 3a), while 60 min of deposition gave a larger thickness of the shell layer. The morphology was not changed after annealing as in the case of TiO_2_ deposited by PLD (Figure S3a,b). On the other hand, after 3 h MS deposition of TiO_2_, the ZNRs were almost completely covered with TiO_2_ (Figure S3c,d) and the transparency of the layer was not preserved. The STEM image of ZnO@TiO_2_ prepared in a deposition time of 1 h (Figure 3c) shows a layer of TiO_2_ that is thicker on the upper side of the nanorods, similar to the samples prepared by PLD. EDS measurements (Table 1) indicate that 1 h of deposition gives a similar atomic percentage of TiO_2_ as a deposition with PLD using 7500 pulses in a vacuum. Although SEM observations indicated a smooth TiO_2_ layer surface, STEM and high angle annular dark field images (HAADF) (Figure S4) indicated that even this smooth surface has a morphology of agglomerated nanoparticles. The HAADF image (Figure S4b) additionally demonstrated that the lower parts of the nanorods, nearer the seed layer, have a much thinner layer of the TiO_2_ then the upper part of the nanorods or did not even contain any TiO_2_. EDS maps (Figure 3d) further confirm that the TiO_2_ layer dominates at the upper part of the ZNR surface, while only a small amount, or none of, the TiO_2_ was deposited between the nanorods.

To study the crystal structure of the TiO_2_ thin layer deposited on ZnO nanorods by MS, Raman spectroscopy was used. Since the Raman spectrum of MS deposited TiO_2_ showed only the band characteristic for ZnO even in the sample prepared by 3 h deposition, it was concluded that the deposited layer is completely amorphous (Figure 3e; spectrum “as deposited”). To obtain the anatase form, superior for further use in photovoltaics, the samples were heated at evaluated temperatures. After heating at 400 °C, even in sample prepared by only 30 min of MS deposition, the Raman bands characteristic for anatase were observed (Figure 3e; spectrum 30 min/400 °C). However, heating to the temperature of 450 °C induced the start of the phase transition to rutile, which was probably the consequence of small particles of TiO_2_, as discussed before for samples prepared by PLD.

To determine the distribution of TiO_2_ on the surface of the ZNRs, GI-XRD at different angles of incidence was applied (Figure 3f). The results indicated that TiO_2_ is thicker at the surface of the ZNRs since the intensity of the anatase peak is larger at small incidence angles. The most intensive peak characteristic for rutile was not observed, which is in agreement with the Raman spectroscopy results.

#### 2.1.4. TiO_2_ Shell Layer Prepared by Chemical Deposition

**Figure 4:**
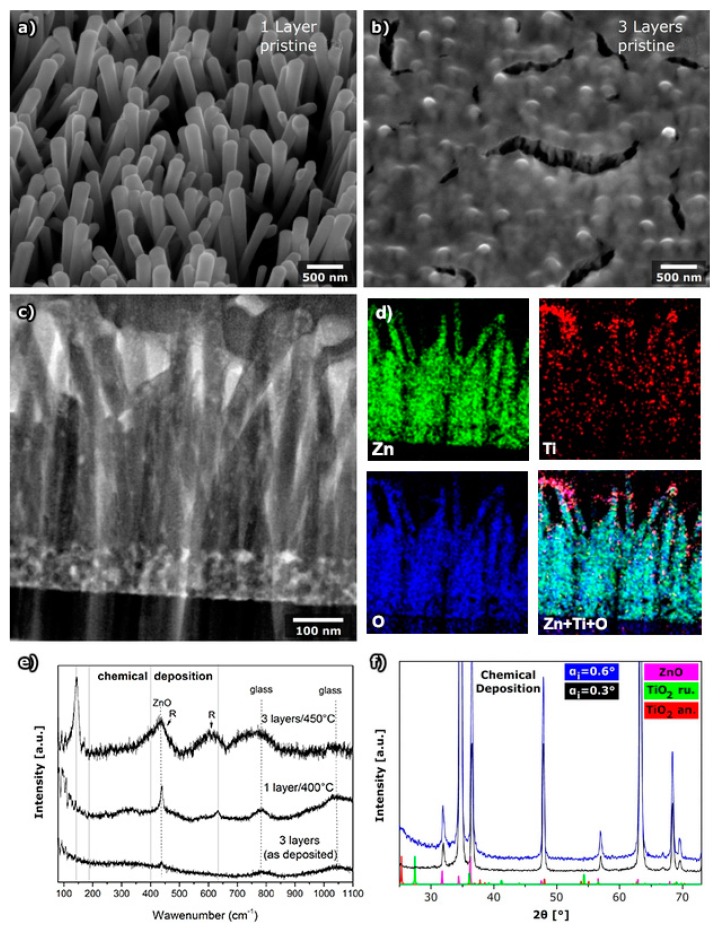
ZnO nanorods with a layer of TiO_2_ deposited by chemical method: (**a**) SEM image after deposition of one layer, (**b**) SEM image after deposition of 3 layers, (**c**) STEM dark field image of a cross section of the sample with one layer, (**d**) EDS maps of zinc, titanium, oxygen, and the distribution of all the elements at nanorods, (**e**) Raman spectra for a sample with 3 deposited layers as deposited, a sample with 1 layer after annealing at 400 °C, and with 3 layers after annealing at 450 °C (vertical lines denote position of anatase bands, bands of ZnO, rutile (R) and glass are also denoted) and (**f**) grazing incidence XRD of the sample with one layer of TiO_2_.

With the aim to obtain the ZnO@TiO_2_ core shell nanorod array using only chemical methods, the TiO_2_ layer was prepared by spin coating of the sol–gel solution and post-deposition annealing at 450 ˚C. The deposition was repeated either one or three times with drying between depositions. The morphology of the nanorods was completely preserved after one deposited layer (Figure 4a), while SEM images of the 3 layers of TiO_2_ deposited on ZNR show a completely covered array with only some cracks in a thick layer (Figure 4b). The EDS measurements indicated the successful deposition of TiO_2_ even if only one layer is deposited (Table 1), but the atomic percentage of Ti was somewhat smaller than in the case of deposition by PLD and MS.

To obtain further insight into the TiO_2_ layer chemically deposited on ZNRs, the cross section of ZnO@TiO_2_ core–shell nanorod array was prepared and recorded by STEM (Figure 4c). STEM images, especially dark field STEM, clearly indicated that the layer of TiO_2_ formed by chemical deposition is much thinner than layers prepared by PDL or MS, but also the thickness of the layer is continuous along the nanorods. This can be explained by the nature of the preparation technique where the prepared sol–gel solution is spin coated on the surface of the ZnO nanorod array, so due to the liquid nature, the substance can be incorporated between the nanorods before crystallization.

The shape of TiO_2_ on ZNR was additionally studied for samples with one deposited layer using EDS mapping measurements (Figure 4d). The measurements were done at the part of the sample shown in dark and bright field images (Figure 4c). The EDS map of Ti indicates that TiO_2_ is homogenously distributed along the ZnO nanorods (Figure 4d).

Since the obtained layer of TiO_2_ was very thin, the Raman spectrum of the one-layer sample (heated at 450 °C) shows bands of ZnO and glass substrate, while bands characteristic for TiO_2_ anatase have very small intensity and are almost completely drowned in the background (Figure 4e). Bands characteristic for TiO_2_ were only observed by Raman spectroscopy for the sample prepared by deposition of 3 layers of TiO_2_, heated at 450 °C, where, beside anatase, some rutile TiO_2_ was also present (Figure 4e). Due to the very thin TiO_2_ layer, the GI-XRD method showed no TiO_2_ peaks (Figure 4f).

### 2.2. Elecrical Properties

**Figure 5:**
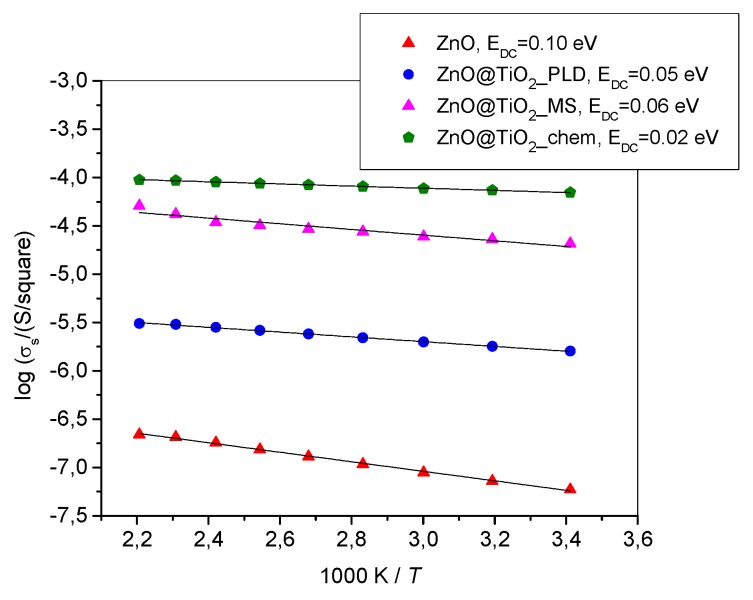
Arrhenius plots of the temperature dependence of the surface conductivity for ZnO nanorods and ZnO@TiO_2_ core–shell nanorods prepared by different deposition procedures. Values of DC activation energy for all measured samples are emphasized.

The surface conductivity of all the samples exhibits Arrhenius temperature dependence, as is shown in Figure 5. The results are shown only for the samples having highest conductivity among samples prepared by specific deposition method. Results presented in Figure 5 are for the samples prepared by PLD in a vacuum by 7500 pulses, 30 min DC reactive MS, and single-layer TiO_2_ prepared by chemical deposition. All ZnO@TiO_2_ core–shell nanorod arrays prepared by different deposition procedures have significantly higher surface conductivity than pure ZnO nanorods. Also, the sample with spin-coating chemically deposited TiO_2_ results in the highest surface conductivity which can be directly related to morphology, i.e. a smooth layer of TiO_2_ that uniformly covers ZnO nanorods (Figure 4). Clearly, such a homogenous TiO_2_ layer allows easy and undisturbed electron transport, leading to higher conductivity.

Surface conductivity spectra at different temperatures for a ZnO@TiO_2_ nanorod array prepared by spin coating one layer of chemically prepared TiO_2_ (Figure S5) show that at each temperature the conductivity is frequency independent. The obtained conductivity plateaus, corresponding to DC surface conductivities, are typical for the fast electronic transport. Similar conductivity spectra were obtained for all samples in this study.

The activation energy for conductivity, *E*_DC_, for each sample was determined from the slope of log*σ*_s_ vs. 1/*T* using the equation

log*σ*_s_ = *σ*_0_exp(−*E*_DC_/*k*_B_*T*) (1)

where *σ*_s_ is the surface conductivity, *σ*_0_ is the pre exponent, *k*_B_ is the Boltzmann constant and *T* is the temperature (K). As expected, the activation energy shows the opposite trend to the changes in the surface conductivity of the samples.

### 2.3. Optical Properties

**Figure 6:**
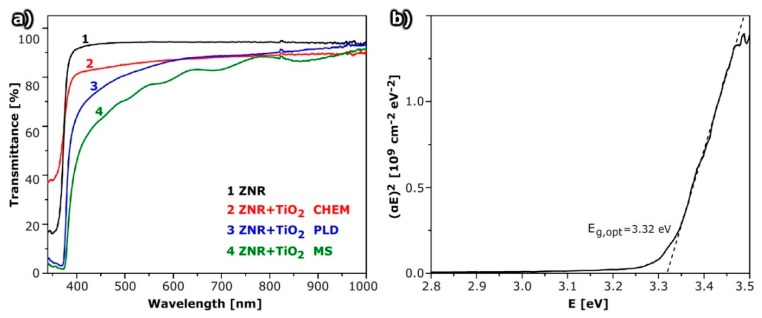
(**a**) Transmittance of ZnO@TiO_2_ nanorods array with a TiO_2_ layer deposited by chemical method (chem), pulsed laser deposition (PLD) in a vacuum using 7500 pulses and magnetron sputtering (MS) for 30 min. (**b**) Determination of the band gap of ZNRs from plot of (*αE*)^2^ versus energy of photons *E*; the line represents fit to the data according to Equation (2) for value *n* = 1/2.

The transmittance of deposited thin films was measured by integration sphere to include the direct and scattered parts of transmitted light. The results for a bare ZnO nanorod array on glass substrate and those covered with TiO_2_ deposited by PLD, MS, and chemical method, are plotted in Figure 6. Samples compared in Figure 6 are those having the best electrical properties among the samples prepared by a specific deposition method.

Figure 6a shows the transmittance of ZnO@TiO_2_ deposited by all three methods—PLD, MS and chemical deposition. The total amount of deposited TiO_2_ is nearly the same for all used methods and the difference between them reveals the variation in structural properties and film morphology. The transmittance of ZnO@TiO_2_ NR arrays is substantially lower than of ZNR due to absorption and possibly internal light scattering in the TiO_2_ layer. The effect is more pronounced for shorter wavelengths, i.e. in the UV part of the spectrum. TiO_2_ deposited by chemical methods forms a continuous film and transmittance is the highest if we compare this result with the results for other deposition methods (Figure 6).

If the transmittance of a ZnO@TiO_2_ NR array for samples with TiO_2_ deposited by PLD is compared, it is observed that the transmittance for a sample deposited in a vacuum is lower than that of samples deposited in an Ar atmosphere. This is due to the higher density and thickness of the TiO_2_ layer, which is consistent with the results of structural measurements (Figure S6). The transmittance of a ZnO@TiO_2_ NR array prepared by MS depends on the deposition time (Figure S7).

The optical gaps, *E*_g,opt_, of ZnO@TiO_2_ NR arrays and ZnO NR thin films were estimated from transmittance as shown in Figure 6b where an example of band gap determination is shown for the case of bare ZNRs. The results were obtained assuming similar behavior as for pure bulk materials, which can be done using the Tauc, Davis and Mott model [30,31]:

*αhν* = *A* (*hν* − *E*_g,opt_)*^n^* (2)

where *A* is a constant and *n* depends on type of optical transition; *n* = 1/2 for direct and 2/3 for indirect transition. For all our films, the direct transition model fits experimental data, and all composite samples have an optical gap around 3.25 eV. Therefore, the optical gap of composites was somewhat smaller than for ZNRs, which is a favorable characteristic for lowering the UV component of the spectrum.

The lower absorbance in the UV part of the spectrum could be beneficial for the application of the composites in photovoltaics since it would protect the active layer. The estimated values for *E*_g,opt_ for composite materials were slightly above values expected from literature values, assuming that the conduction band is not empty (the Burstein–Moss effect) which is consistent with electrical measurements that show relatively low activation energy.

## 3. Discussion

**Figure 7:**
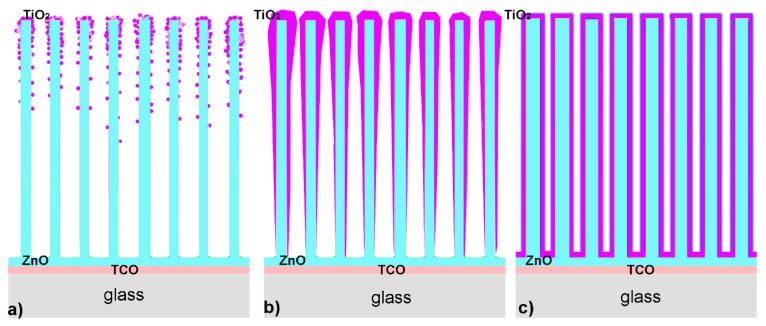
The schematic view of obtained TiO_2_ layers with different morphologies: (**a**) TiO_2_ formed in the shape of nanoparticles having different sizes, (**b**) a smooth layer of TiO_2_ but thicker on the upper side of the ZnO NR, and (**c**) a smooth layer of TiO_2_ that homogenously covers the ZnO NR.

Since it is known from literature [6,7,14,15,16,17,18] that ZnO@TiO_2_ core–shell nanostructures could serve as an advantageous electron transport layer in perovskite, polymer, and dye sensitized solar cells, influence of the morphology and structure on electrical and optical properties, that are crucial for photovoltaic application, were studied. To obtain ZnO@TiO_2_ core–shell nanostructures with diverse structural properties, different deposition techniques were applied for the preparation of the shell layer, and the deposition parameters were additionally varied. In contrast to known literature where usually one chemical method was applied for the preparation of the TiO_2_ shell layer on ZnO, in this study the focus was on the influence of different preparation methods (two physical and one chemical) on properties of the core–shell structure. Depending on the deposition techniques, we obtained 3 different morphologies of TiO_2_ shell layers (Figure 7). As was expected, the morphology of the shell layer affected the electrical and optical properties responsible for future application in photovoltaics. The main morphologies of TiO_2_ layers: (1) nanoparticles or nanoaggregates on the surface of nanorods schematically represented by Figure 7a, (2) a layer that preferably covers the upper part of nanorods, thicker at the top of nanorods, represented by Figure 7b, and (3) a layer of TiO_2_ that is homogenously distributed along the whole length of nanorods, represented by Figure 7c. The first morphology was observed for ZnO@TiO_2_ core shell nanorods obtained by PLD in argon. The second morphology was characteristic for TiO_2_ layers deposited by PLD in a vacuum or by magnetron sputtering, while the homogenously covered nanorods were prepared by chemical deposition.

The preparation parameters of each method influenced the thickness of the TiO_2_ layer. Thicker layers were obtained by more laser pulses for PLD deposition, for longer time of MS deposition, or by more layers applied by spin coating. For the larger thickness of TiO_2_ layers prepared by MS or chemical deposition, the nanorods were completely cluttered with TiO_2_ (Figure S3 and Figure 4b). Since for thicker layers the optical properties are poor due to lower transparency, the optical and electrical properties were studied only for the samples with thinner TiO_2_ layers.

The structural study indicated that as deposited layers were amorphous, so all the layers were additionally annealed after deposition to obtain crystallinity. Since anatase TiO_2_ has better optical properties, the heating temperature was chosen to obtain a pure anatase structure of the shell layer. It was observed that a relatively low heating temperature of only 400 °C should be applied to obtain an anatase structure of the TiO_2_ shell layer, since at 450 °C the partial phase transition to rutile was already observed, although the phase transition to rutile is around 1000 °C for large monocrystals of TiO_2_. The low temperature of phase transition was explained by small particles/clusters or very thin layers of amorphous TiO_2_ that accelerate the phase transition even at so low a temperature as 400 °C.

The impedance spectroscopy measurements show pure electron conductivity that was considerably larger in formed nanostructures than in the bare ZnO nanorod array or bare TiO_2_ layer. The highest conductivity and the lowest activation energy among the studied samples were observed for the sample with a chemically deposited TiO_2_ shell. This result can be explained by the homogenousTiO_2_ layer at the nanorod surface. The homogeneity was achieved, as during chemical deposition the precursor in the form of solution was spin coated at the surface of ZnO NR and can penetrate between nanorods, so that after heating, the formed TiO_2_ was distributed along the nanorods.

Optical measurements show the absorption in the UV part of the spectrum for all the morphologies, but the absorption in the visible part depends on the morphology of the TiO_2_ layer. The highest transparency among the studied samples was achieved for samples formed by chemical deposition, which can be explained both by the thickness of the layer as well as the homogeneity.

In comparison with data obtained for similar nanostructures presented in literature [32], our samples showed higher transmission and better conductivity. Ahmadi et al. [32] prepared ZnO/TiO_2_ composite core–shell nanorod arrays by sol–gel process and dip coating, and measured transmittance that was lower than 60% in the visible part of the spectrum and even lower than 40% in the spectral region under 450 nm; while in our study, we obtained more than 80% transmittance. Resistivity of our core–shell structures varied between 10^4^ and 10^6^ Ω (Figure 5). This value corresponds to the specific resistivity in the range between 1 and 10 Ωcm, which is much lower than in [32]. On the other hand, if we compare our results of optical measurements obtained for arrays of 1-dimensional ZnO@TiO_2_ core–shell nanorod arrays with results obtained from compact TiO_2_/ZnO composite thin films studied by Giannakopoulou et al. [33], it can be seen that our transmittance in the visible part of the solar spectrum is comparable to or slightly lower than that obtained from compact TiO_2_/ZnO thin films [33]. However, their thin films are considerably thinner (thicknesses are approximately 60–145 nm) in comparison to height of our ZnO@TiO_2_ core–shell nanorods that form arrays (>300 nm), thus indicating the much better optical properties of our core–shell nanorod arrays.

We can compare the optical properties of the obtained ZnO@TiO_2_ core–shell nanorods studied in this work with the optical properties obtained by Zou et al. [34] for ZnO/V_2_O_5_ composite core–shell nanorod arrays with similar morphologies as our samples. It could be seen that for their as-prepared samples, the absorption edge is shifted to a larger wavelength, even to around 500 nm, while for samples annealed at 500 °C, considerable absorption of the light was observed in the completely visible region. Therefore, they have suggested the use of these nanocomposites for photocatalytic applications with visible light or natural sunlight [34].

The prominent electron transport and optical properties of our ZnO@TiO_2_ core–shell nanorod arrays seems promising for application as an electron transport layer in perovskite and dye-sensitized solar cells. Since we have found appropriate preparation parameters of the studied deposition methods to obtain preferable electrical and optical properties for photovoltaic applications, our next step will be the assembling of photovoltaics using the studied ZnO@TiO_2_ core–shell nanorod arrays and a study of their performance in correlation with the TiO_2_ shell layer preparation method types and parameters.

## 4. Materials and Methods

The following materials and chemicals were used: ITO glass slides (20 × 15 mm, Ossila), Ti and TiO_2_ targets (for magnetron and pulsed laser depositions), deionized water (Milipore), acetone, ethanol (EtOH, aps.), zinc acetate dihydrate (ZA), zinc nitrate hexahydrate (ZN), hexamethylentetramine (HMT), titanium isopropoxide (TiPr), ethylene glycol monomethyl ether (EGME), monoethanolamine (MEA). All of the materials were analytical grade and used as received without further purification.

ZnO nanorods (ZNRs) were prepared in two steps. The first step was deposition of a ZnO seed layer by spin coating a solution of 0.25 M ZA in EtOH and MEA on previously cleaned (acetone, ethanol, and water in ultrasound for 10 min each, followed by UV ozone cleaner for 30 min) ITO substrates. After the deposition of the seed layer, the substrates were immersed in an aqueous solution of 0.125 M zinc nitrate hexahydrate and 0.125 M hexamethylenetetramine at 92 °C for 3 h. For electrical measurement, the ZnO seed layer and nanorods were grown directly on a glass (or quartz) surface to avoid artefacts in the conductivity measurement due to short circuits possibly caused by the mounting of contact electrodes.

ZNRs were further covered with TiO_2_ using two different physical deposition procedures and one chemical procedure. The evaluated procedures were: pulsed laser deposition (PLD), DC reactive magnetron sputtering, and spin coating of a chemically prepared precursor followed by annealing.

**Figure 8:**
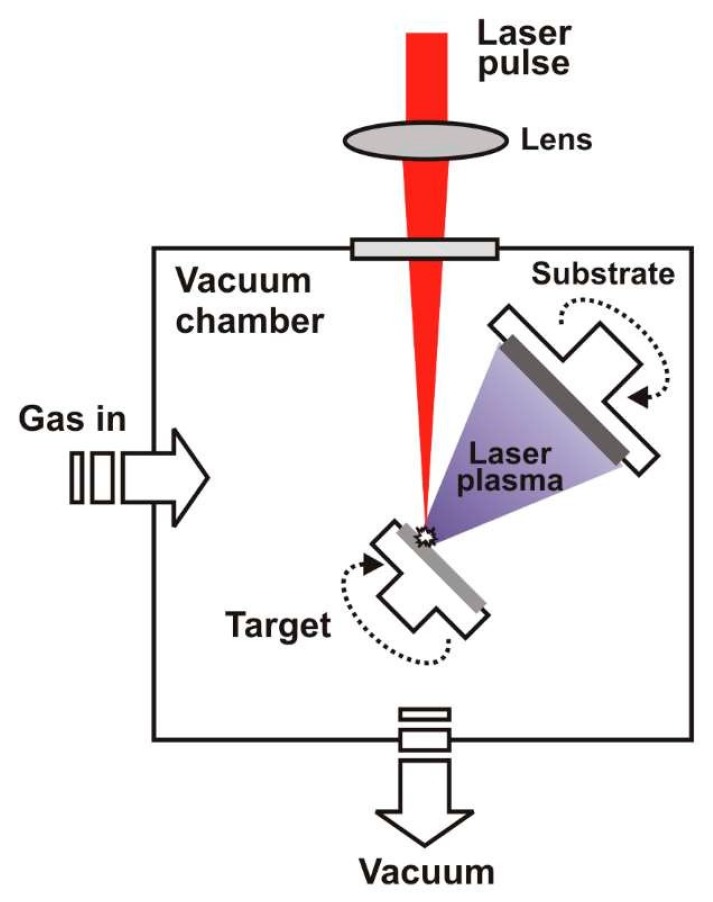
Schematic view of pulsed laser deposition device setup.

Pulsed laser deposition of TiO_2_ thin films on the surface of ZNRs was done by using a Nd:YAG laser. The experimental setup is shown in Figure 8. Laser parameters were: wavelength 1064 nm, pulse duration 4 ns, repetition rate 5 Hz, and output energy 340 mJ. Laser pulse energy in front of target was 150 mJ while laser pulses were focused on the target surface which yields a fluence of 20 J/cm^2^. The distance between the target and substrate was 4 cm. The target surface was parallel to the substrate and inclined by 45° with respect to the impinging laser pulses. Both the target holder and the substrate were kept on floating potential and at room temperature during deposition and were rotated to avoid the drilling of the target and to increase the homogeneity of the deposited films. Films were deposited using 5000 and 7500 laser pulses. Deposition was performed either in a vacuum (pressure < 10^−3^ mbar) or in 30 Pa of argon. After deposition, annealing at high temperature should be done to obtain crystallinity. The temperature of annealing was varied to obtain a pure anatase structure of TiO_2_ and to avoid crystallization of the rutile TiO_2_, so the temperatures of 400, 450, and 500 °C were applied.

Preparation of TiO_2_ thin films on ZNRs by DC reactive magnetron sputtering was done using a 2″ Ti 99.999% target, an Ar + O_2_ mixture as working gas and deposition parameters as listed in Table 2. Depositions were done at room temperature. However, because of the interaction with plasma, substrate temperature was increased during the deposition to 60 °C. To obtain crystalline anatase, TiO_2_ annealing was applied at temperatures between 400 and 500 °C.

The chemical deposition of TiO_2_ thin films on ZnO NRs was done by spin coating (4000 rpm, 30 s) the sol–gel solution of 0.125 M TiO_2_ (TiPr in EGME, MEA, and EtOH) and annealing at 400 or 450 °C after deposition.

The morphology of the samples was studied by scanning electron microscopy with a field emission gun (FEG-SEM) using JEOL JSM-700F (JEOL, Tokyo, Japan). The crystal structure on nanoscale was studied by transmission electron microscopy (TEM) using a JEOL-ARM300 microscope (JEOL, Tokyo, Japan) in TEM mode and high angle annular dark field (HAADF) mode.

The structural phase of TiO_2_ thin films was studied by confocal micro-Raman spectroscopy, using a Jobin Yvon T64000 (HORIBA Jobin Yvon GmbH, Bensheim, Germany) with solid state laser operated at 532.5 nm for excitation. The objective with 50x magnification and large working distance was used. The power of the laser was 2, 7, or 20 mW, depending on the sample. It was optimized to avoid heating in the focus of the laser beam that could induce the phase transition of TiO_2_.

GIXRD were obtained using synchrotron X-ray radiation at the MCX beamline at synchrotron Elettra (Trieste, Italy) in grazing incidence geometry with the wavelength of 0.155 nm (8.00 keV) [35]. The diffraction patterns were obtained at several values of the angle of incidence, slightly above the critical angle for total external reflection (α_i_ = 0.2°, 0.3°, 0.6°), in order to probe at different depths below the sample surface. The scattered intensity was collected in 2θ angular range 10°–75° with a 2θ step size of 0.05°.

The optical transparency was measured by UV–vis spectroscopy, using a Xe 150 W light source and an Ocean Optics HR4000 spectrometer (Ocean Optics, Largo, FL, USA) equipped with an integration sphere.

For electrical characterization, impedance spectroscopy (Novocontrol Alpha-A Dielectric Spectrometer, Novocontrol Technologies, Montabaur, Germany) was used. The complex impedance was measured in the frequency range from 0.01 to 1 MHz at a voltage of 10 mV and over a temperature range from 293 and 453 (± 0.5) K. A frequency sweep at each temperature was repeated twice. For electrical contacts, silver paint pads, 2 mm in length and separated by 2 mm, were placed on the surface of the sample.

## 5. Conclusions

ZnO@TiO_2_ core–shell nanorod arrays were prepared using a two-step process; ZnO nanorods as cores were prepared by a wet chemistry procedure, while TiO_2_ shells were deposited by three different methods. As physical methods, pulsed laser deposition or magnetron sputtering were used; while as a chemical method, spin coating followed by heating of a chemically prepared TiO_2_ precursor.

TiO_2_ layer morphology depends on the used deposition method and deposition parameters. We obtained three different TiO_2_ morphologies: (1) separated nanoparticles and agglomerates (prepared by PLD in Ar), (2) a layer with larger thickness at the top part of the nanorods (prepared by PLD in vacuum and MS), and (3) a homogenous thin layer along the whole length of nanorods (chemical deposition). All the layers were additionally heated after deposition to obtain crystallinity.The relatively low heating temperature of 400 °C should be applied to obtain anatase structures of the TiO_2_ shells, since at 450 °C the phase transition to rutile was already observed.

The impedance spectroscopy measurements show pure electron conductivity that was considerably larger in the formed nanostructures then in a bare ZnO nanorod array or bare TiO_2_ layer. The activation energies were low and depend on the structural and morphological parameters of the TiO_2_ shell. The highest conductivity and the lowest activation energy among the studied samples were observed for the sample with a chemically deposited TiO_2_ shell that has homogenously deposited TiO_2_.

Optical measurements show a significant absorption in the UV part of the spectrum for all the morphologies, but the absorption in the visible part depends on the morphology of the TiO_2_ layer. The best transparency among the studied samples is achieved for the samples formed by chemical deposition that have homogenous distribution of TiO_2_ in the shell layer.

By comparing the optical and electrical properties of the structures, it can be concluded that for the use of ZnO@TiO_2_ core–shell nanorod arrays for photovoltaic applications, the method of preparation and the preparation parameters should be carefully chosen to obtain applicable optical and electrical properties.

## Figures and Tables

**Table 1:** Results of EDS measurements at samples prepared by different deposition routes.

Sample/Preparation Procedure	Zn L (at%)	Ti K (at%)	O K (at%)	Si K (at%)
ZnO@TiO_2_ – PLD (Ar, 7500 p)	44.6	3.0	49.2	3.2
ZnO@TiO_2_ – PLD (vacuum, 7500 p)	23.8	8.8	56.8	10.6
ZnO@TiO_2_ – MS (30 min)	37.5	5.0	54.0	3.5
ZnO@TiO_2_ – MS (1 h)	37.8	7.9	53.3	1.0
ZnO@TiO_2_ – MS (3 h)	18.6	19.2	61.6	0.6
ZnO@TiO_2_ – chem (1 layer)	40.7	1.3	51.2	6.8
ZnO@TiO_2_ – chem (3 layers)	28.8	4.2	54.8	12.2

**Table 2:** Magnetron sputtering deposition parameters used for the TiO_2_ thin film on ZNR deposition.

Parameter	Value
Base pressure	7 × 10^−7^ mbar
Ar/O_2_ flow rate ratio	5 × 10^−3^ mbar
Discharge power	100 W
Discharge current	200 mA
Target to substrate distance	100 mm

## Supplementary Materials

The following are available online at https://www.mdpi.com/1420-3049/24/21/3965/s1, Figure S1: Scanning electron microscopy images of ZnO NR (surface view) with layer of TiO_2_ deposited by PLD: (a) using 7500 pulses of laser (5Hz) in vacuum, pristine as deposited, (b) using 7500 pulses (5Hz) in Ar, pristine as deposited (c) using 7500 pulses in Ar after heating in air at 400 °C, (d) using 5000 pulses (5Hz) in Ar after heating in air at 400 °C surface view., Figure S2: Raman spectra of TiO_2_ deposited by PLD: (a) in vacuum and (b) in 30 Pa argon. Full lines denoted the positions of anatase Raman bands, while the number of laser pulses and annealing temperature are denoted above spectra., Figure S3: ZnO@TiO_2_ core-shell nanorods with TiO_2_ layer prepared by DC reactive magnetron sputtering: (a) 1 h deposition, pristine, (b) 1 h deposition, after anealling at 400 °C, (c) 3 h deposition, pristine, top view, and (d) 3 h deposition, pristine, side view, Figure S4: The cross section ZnO@TiO_2_ core-shell nanorods with TiO_2_ layer prepared by 1 h of DC reactive magnetron sputtering: (a) bright field STEM, (b) HAADF., Figure S5: Conductivity spectra at different temperatures for ZNR@TiO_2_ prepared by spin coating deposition of chemically prepared TiO_2_., Figure S6: UV-Vis transmittance results of ZnO@TiO_2_ NR array with TiO_2_ deposited by PLD measured by integration sphere, Figure S7: Transmittance of ZnO@TiO_2_ NR array with TiO_2_ is deposited by magnetron sputtering and thin TiO_2_ films deposited on glass under same conditions.
