# Supplementary material for: ZnO@TiO2 Core Shell Nanorod Arrays with Tailored Structural, Electrical, and Optical Properties for Photovoltaic Application
Source: Molecules. 2019 Nov 1;24(21):3965. doi: 10.3390/molecules24213965 (PMC6864808; doi:10.3390/molecules24213965)
Supplement: Supplementary file 1 [file molecules-24-03965-s001.pdf]

Supplementary Materials:

S1. Structural results

S1.1. TiO_2 shell layer prepared by pulsed laser deposition (PLD)

In sample of ZnO nanorods with deposited TiO_2 layer using 7500 pulses of laser (5Hz) in vacuum formed smooth layer of TiO_2 at the surface (Figure S1 a), while in Ar atmosphere (30 Pa), TiO_2 formed larger nanoparticles and clusters on the surface of ZnO NR (Figure S1 b). After heating at 400 °C, the morphology of the TiO_2 layer was not changed (Figure S1 c). The number of laser pulses influenced the shape of upper part of nanorods, so by using the lower number of pulses (5000) the top of the nanorods show minor extending (Figure S1 d).

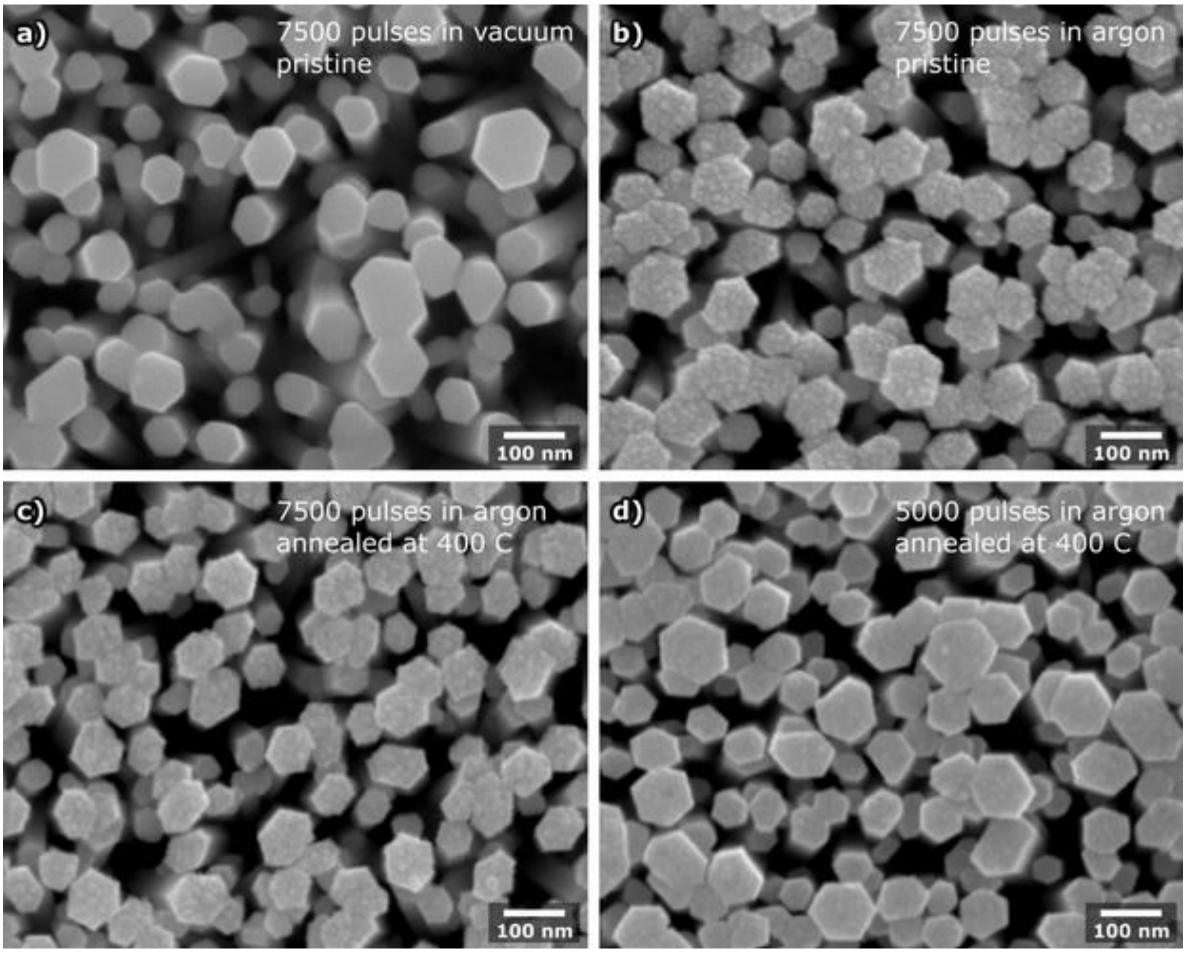

Figure S1: Scanning electron microscopy images of ZnO NR (surface view) with layer of TiO_2 deposited by PLD: a) using 7500 pulses of laser (5Hz) in vacuum, pristine as deposited, b) using 7500 pulses (5Hz) in Ar, pristine as deposited c) using 7500 pulses in Ar after heating in air at 400 °C, d) using 5000 pulses (5Hz) in Ar after heating in air at 400 °C surface view.

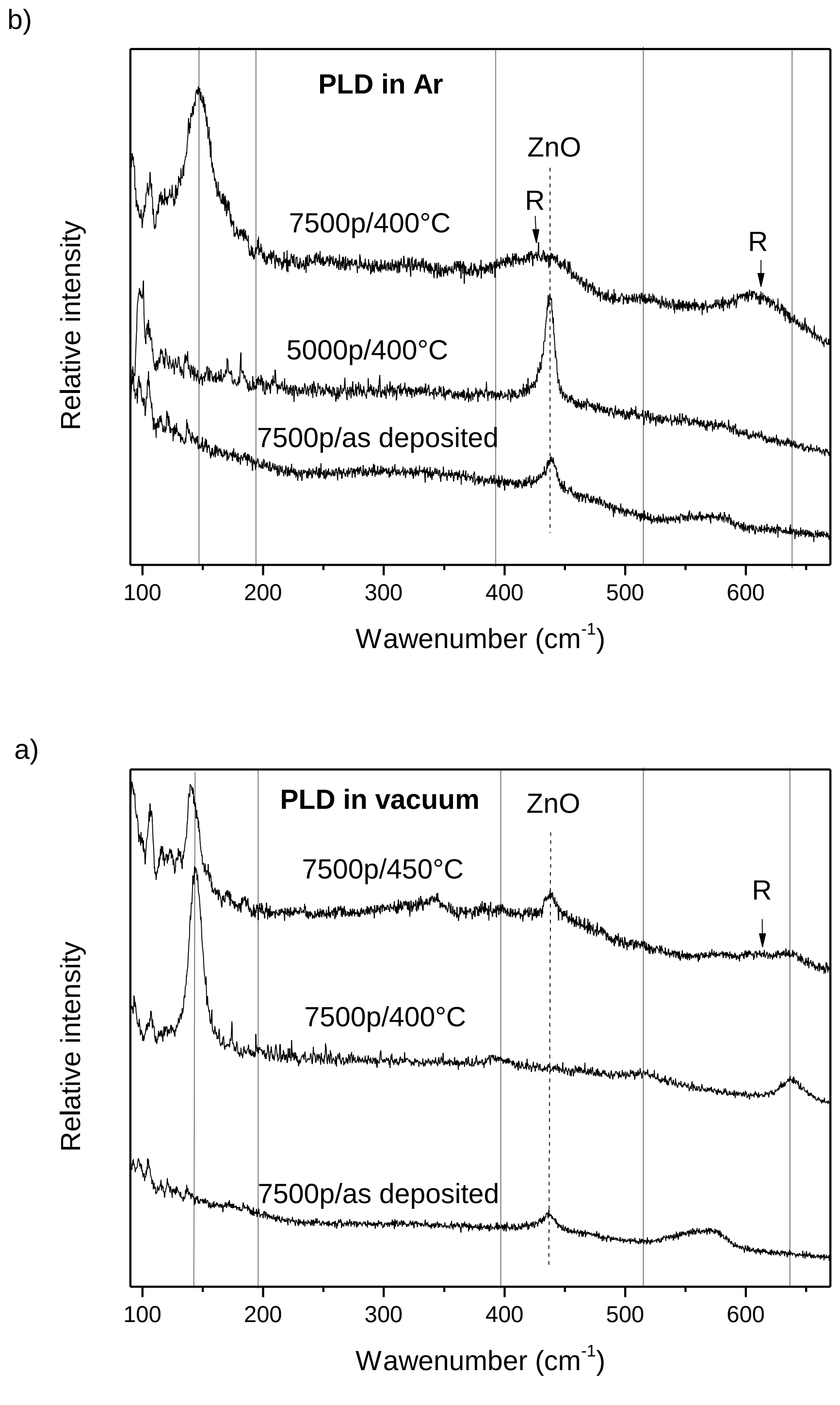

Figure S2: Raman spectra of TiO_2 deposited by PLD: a) in vacuum and b) in 30 Pa argon. Full lines denoted the positions of anatase Raman bands, while the number of laser pulses and annealing temperature are denoted above spectra.

S1.2. TiO_2 shell layer prepared by magnetron sputtering

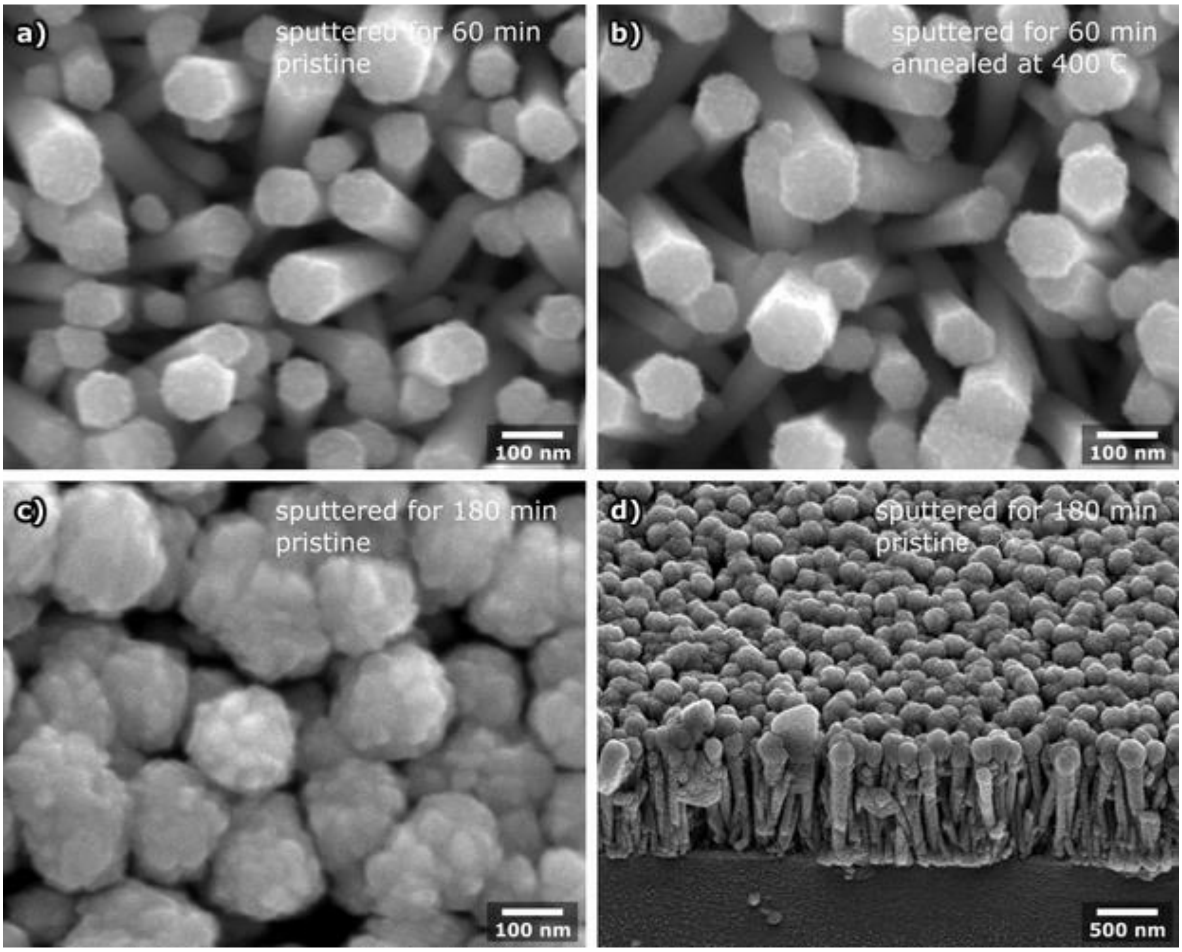

Figure S3: ZnO@TiO_2 core-shell nanorods with TiO_2 layer prepared by DC reactive magnetron sputtering: a) 1 h deposition, pristine, b) 1 h deposition, after anealling at 400 °C, c) 3 h deposition, pristine, top view, and d) 3 h deposition, pristine, side view

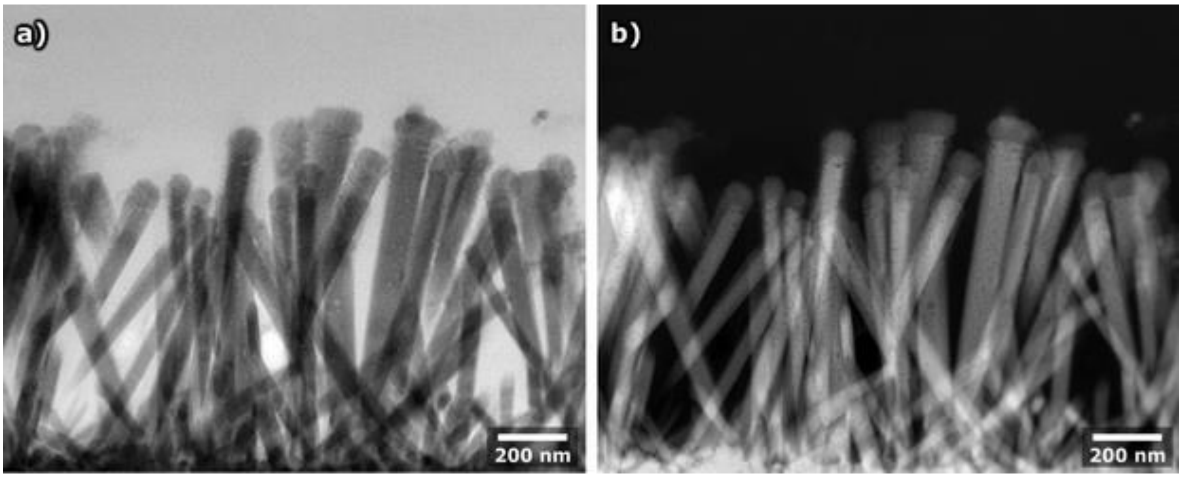

Figure S4: The cross section ZnO@TiO_2 core-shell nanorods with TiO_2 layer prepared by 1 h of DC reactive magnetron sputtering: a) bright field STEM, b) HAADF.

S2. Electrical properties

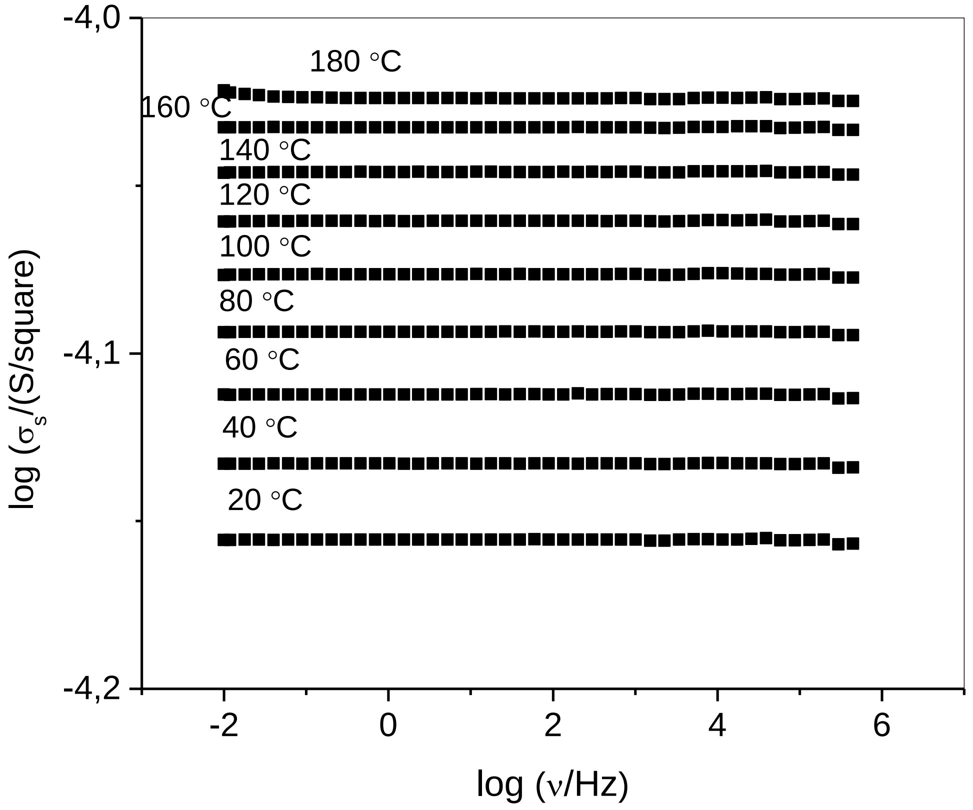

Figure S5: Conductivity spectra at different temperatures for ZNR@TiO_2 prepared by spin coating deposition of chemically prepared TiO_2.

S3. Optical properties

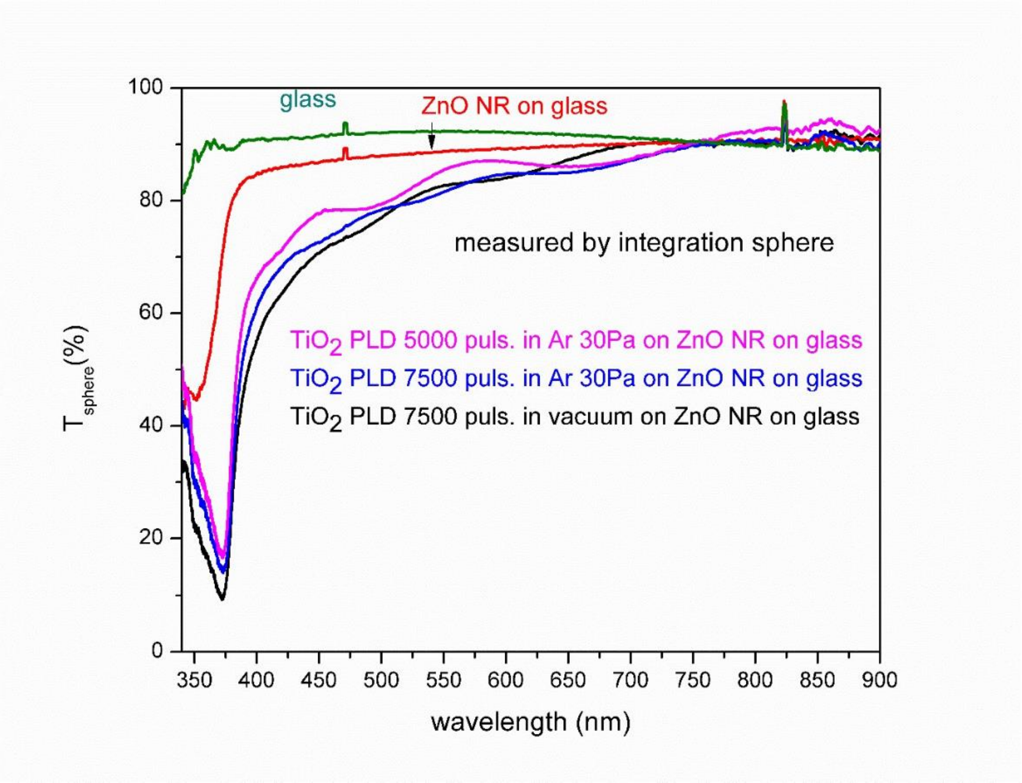

Figure S6: UV-Vis transmittance results of ZnO@TiO_2 NR array with TiO_2 deposited by PLD measured by integration sphere.

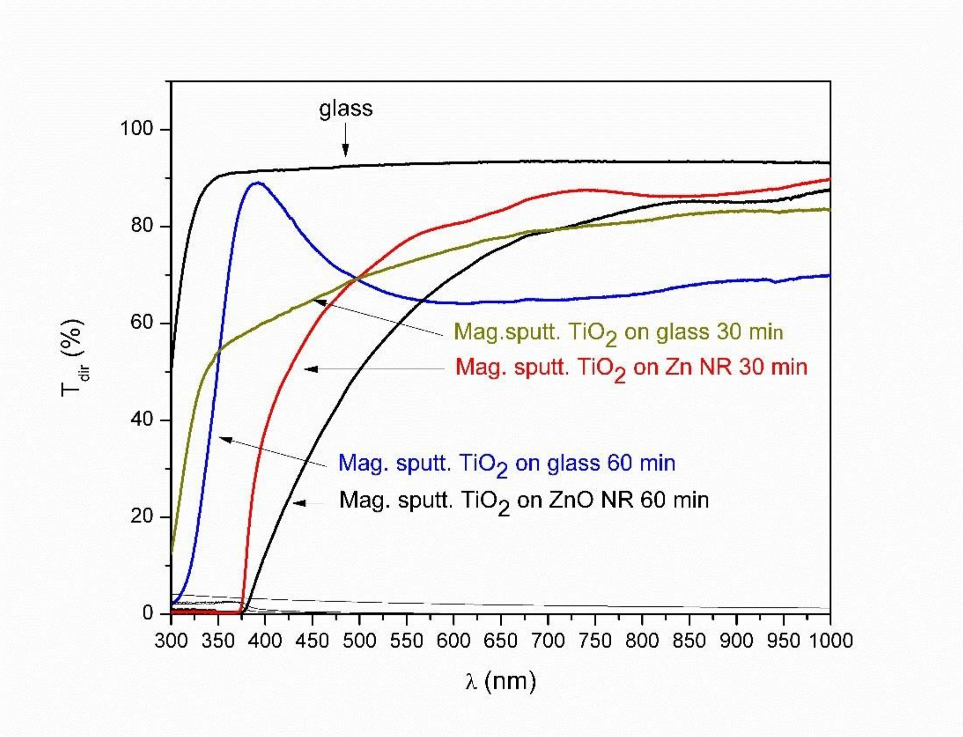

Figure S7: Transmittance of ZnO@TiO_2 NR array with TiO_2 is deposited by magnetron sputtering and thin TiO_2 films deposited on glass under same conditions.
